# DOES COGNITIVE RESERVE MODERATE THE RELATIONSHIP BETWEEN STROKE SEVERITY AND COGNITIVE FUNCTION?

**DOI:** 10.1093/geroni/igae098.1013

**Published:** 2024-12-31

**Authors:** Yinuo Ou, Yalin Lv, Hui Wei, Yanrong Zhang, Juan Li

**Affiliations:** Huashan Hospital Fudan University, National Center for Neurological Disorders of China, Shanghai, Shanghai, China (People’s Republic); Huashan Hospital Fudan University, Shanghai, Shanghai, China (People’s Republic); Huashan Hospital Fudan University, National Center for Neurological Disorders of China, Shanghai, Shanghai, China (People’s Republic); Huashan Hospital Fudan University, National Center for Neurological Disorders of China, shanghai, Shanghai, China (People’s Republic); Huashan Hospital Fudan University, Shanghai, Shanghai, China (People’s Republic)

## Abstract

Cognitive decline is common after stroke. This study aimed to explore the moderating effect of cognitive reserve on the relationship between stroke severity and long-term cognitive function.A longitudinal survey of patients with acute ischemic stroke (AIS) was conducted in 2023 at four stroke centers in Nanjing and Shanghai, China. A total of 371 eligible patients were interviewed at acute stage and re-interviewed at 3 and 6 months after onset. Stroke severity was assessed using The National Institutes of Health Stroke Scale(NIHSS) at admission. Cognitive reserve was assessed by Cognitive Reserve Index questionnaire (CRIq) within 7 days after stroke onset. Cognitive function was assessed by Montreal Cognitive Assessment-Changsha Version(MoCA-CS) at each wave. A series of general linear mixed models were applied to test the moderating effect of cognitive reserve.The study found that cognitive function improved over time in all patients after stroke(β=1.314, p<0.001). The cognitive function of patients with higher NIHSS score was worse than those with lower NIHSS score(β=-1.315, p<0.01).Patients with a higher level of cognitive reserve had better cognitive function after stroke than those with a lower level of cognitive reserve(β=0.048, p<0.01). The interaction between NIHSS and cognitive reserve was statistically significant(β=0.012, p<0.05)after controlling for a covariate[Trial of ORG 10172 in Acute Stroke Treatment (TOAST)].Cognitive reserve can mitigate the impact of stroke burden on long-term cognitive decline in Chinese AIS patients to some extent. Cognitive reserve provides a potential target for the prevention and intervention of cognitive impairment after stroke.

